# Association Between Food Opportunities During the School Day and Selected Dietary Behaviors of Alternative High School Students, Minneapolis/Saint Paul, Minnesota, 2006

**Published:** 2010-12-15

**Authors:** Chrisa Arcan, Martha Y. Kubik, Jayne A. Fulkerson, Cynthia Davey, Mary Story

**Affiliations:** University of Minnesota School of Public Health, Division of Epidemiology and Community Health; University of Minnesota School of Nursing, Minneapolis, Minnesota; University of Minnesota School of Nursing, Minneapolis, Minnesota; University of Minnesota Academic Health Center, Minneapolis, Minnesota; University of Minnesota School of Public Health, Minneapolis, Minnesota

## Abstract

**Introduction:**

Availability of competitive foods in schools has been linked to unhealthful dietary behaviors of students. Little is known about the food environment of alternative high schools, schools that enroll students at risk of academic failure. We examined correlations between food opportunities during the school day and selected dietary behaviors of students attending alternative high schools.

**Methods:**

Baseline data were collected in fall 2006 as part of the Team COOL (Controlling Overweight and Obesity for Life) pilot study, a group randomized obesity prevention trial. Students (n = 145) attending 6 alternative high schools in Minneapolis/Saint Paul, Minnesota, completed a survey on food opportunities during the school day and selected dietary behaviors. We used mixed-model multivariate cross-sectional analysis and adjusted for demographic characteristics to examine associations of interest.

**Results:**

Food opportunities during the school day were positively associated with overall consumption of sugar-sweetened beverages, high-fat foods, and fast-food restaurant use.

**Conclusions:**

Having many food opportunities during the school day at alternative high schools was linked to the consumption of foods and beverages high in sugar and fat and low in nutrients. School-based interventions should focus on changing the food environment in alternative high schools to decrease less healthful eating opportunities and to increase the availability of healthful foods and beverages.

## Introduction

In the United States, more than 95% of children are enrolled in school, where they consume up to half of their total daily energy intake (1). The federally regulated National School Lunch Program (NSLP) is a major source of food at school. However, student participation in the NSLP declines by 27% from elementary to high school because competitive foods from venues such as à la carte programs, snack bars, vending machines, and school stores become more available (2). Furthermore, competitive foods, which are often high in fat, sodium, and added sugars, are not subject to the same nutrition guidelines that apply to foods served in the NSLP (3). Higher availability of competitive foods in schools is associated with higher consumption of these foods and lower consumption of healthful items (2,4,5). In addition, school policies, such as open campus, which allow students to leave school during lunch, may result in students purchasing food from nearby convenience stores and fast-food establishments (4). These policies may further increase intake of high-fat foods because french fries and hamburgers are the most frequently sold items in fast-food restaurants (6). Other food practices, such as allowing students to eat or drink in school hallways and classrooms, have been associated with higher student weight (7).

This study focuses on alternative high schools and their students, a group of youth at risk of academic failure (8). Nationwide, more than half a million students are enrolled in 6,600 public alternative school programs (9). Alternative high schools are more likely than traditional high schools to have high enrollment of minority and low-income students (10). Alternative high school students are also more likely than traditional high school students to engage in health-compromising behaviors (11). Studies have shown a high prevalence of overweight and obesity, unhealthful dietary practices, and low physical activity levels among students attending alternative high schools (12-14).

Few studies have examined the alternative school food environment (15,16). Results from a focus group study of alternative high school students found that students were dissatisfied with the food at school, including the school lunch. Many students report skipping lunch and snacking on vending machine food or purchasing food off-campus (16). Since 2006, public school districts are required to have a written local wellness policy that sets nutrition guidelines for foods and beverages available at school (17). Although it seems that alternative and traditional high schools in the same district would have the same wellness policies, alternative schools may be governed at the school level and their policies may vary from those of traditional schools or may be interpreted differently (8). Therefore, the application of wellness policies, including those that affect the school food environment, may lack uniformity among schools in the same district.

Our aim was to assess correlations between food opportunities during the school day and dietary behaviors, specifically consumption of sugar-sweetened beverages, high-fat foods, and fruits and vegetables, and use of fast-food restaurants among alternative high school students.

## Methods

### Study design

Students were participants in the Team COOL (Controlling Overweight and Obesity for Life) pilot study, a multicomponent diet and physical activity intervention trial to promote healthy weight loss or prevent excess weight gain (12). We contacted 6 alternative public high schools (4 urban and 2 suburban) in the  Minneapolis/Saint Paul metropolitan area that agreed to participate. Data were collected in October and November 2006 before schools were randomly assigned to intervention and control conditions. Additional details about the study design are described elsewhere (12).

### Study population

Student enrollment in the 6 schools varied from 27 to 145 students (mean, 102 students). All students enrolled in the schools were eligible to participate in study measurements, including a survey and measures of height and weight. Thus, there were no inclusion and exclusion criteria for the study participants. Parental consent forms were given to students younger than 18 years. On the day of measurement, trained study staff collected assents from all students and the signed parental consents. The students completed a 76-item survey. Details about the survey have been reported elsewhere (12). (A copy of the survey is available on request from the corresponding author). Students who completed the survey and had their height and weight measured received a $5 gift card. All study procedures were approved by the University of Minnesota's institutional review board.

Across the 6 schools,  145 students completed the baseline survey. Due to the variable nature of student attendance in alternative high schools, the estimated participation rate was based on adjusted attendance, which was derived by multiplying each school's attendance rate for the previous year (2005-2006) with the school's 2006-2007 student enrollment (18) to give an estimated average adjusted attendance of 68 students (range, 16-107). The participation rate across schools was then 36% (range, 18%-100%).

### Measures

**Demographic characteristics**

Sex, age, race/ethnicity, and socioeconomic status (SES) were in the models as potential confounders. We obtained age and sex from school records and asked students to report their race/ethnicity. To ensure adequate sample size for analyses, we combined the categories into white, black, and "other." The "other" category includes the following groups: American Indian or Alaska Native; Asian, including Cambodian, Hmong, Korean, Laotian, and Vietnamese; Hispanic or Latino; and multiethnic non-Hispanic. SES was measured with the question "Do you get free/low-cost lunches at school?" A total of 135 respondents answered yes or no; 10 answered, "I don't know." If the response was missing or "I don't know," the question "Does your family get public assistance (welfare, food stamps, or other assistance?)" was used. Of the 10 who reported "I don't know," 8 reported yes or no to the second question. The new SES variable was dichotomized to yes = lower SES and no = higher SES.

**Dietary behaviors**

The following dietary behaviors are dependent variables and are included in separate models:

**Consumption of regular soda, sports drinks, and other sugar-sweetened beverages.** We asked participants to report the frequency of their consumption of regular soda, sports drinks, and other sugar-sweetened beverages (Kool-Aid, fruit drinks, lemonade, or energy drinks) during the past month (19). Ten response categories ranged from "never" to "5 or more times a day." The data were recoded as times per week and were modeled as a continuous variable. High values indicated more beverage consumption per week.

**High-fat food intake.** We used the 17-item fat screener developed by Block and colleagues (20) to collect students' reported intake of high-fat food. The screener has been previously validated in an adult population and was considered appropriate for use with an older adolescent study sample (20). Examples of high-fat foods included various meats, hot dogs, fried chicken, pizza, whole milk and cheese, french fries, and doughnuts. Five response categories ranged from "1 time a month or less" to "5 or more times a week." Data were recoded to represent times per week and were modeled as a continuous variable. The Cronbach α for the study sample was 0.89. Students whose responses were greater than 3 standard deviations (SD) from the mean were excluded from the analysis (n = 2). Higher values indicated more frequent consumption per week.

**Fruit and vegetable intake.** We used a previously validated 6-item fruit and vegetable screener (21) to collect students' reported frequency of fruit and vegetable intake. The screener was validated in a racially and socioeconomically diverse sample of urban high school students (21). The items included 100% fruit juice, fruits, vegetables, green salad, potatoes (excluding french fries), and carrots. The 6 response categories ranged from "less than once a week" to "5 or more times a day." Data were recoded as daily servings and were modeled as a continuous variable. The Cronbach α for the study sample was 0.85. Students whose responses were greater than 3 SDs from the mean were excluded from the analysis (n = 2). High values indicated more servings per day of fruits and vegetables.

**Fast-food restaurant use**. We asked students to report how many times they ate or drank something from a fast-food restaurant outside of the school day (including weekend days) (19). Six response categories ranged from "never" to "more than 7 times per week." The data were recoded to represent times per week and were modeled as a continuous variable. Higher values indicated more visits to a fast-food restaurant.

### Food opportunities and the school food environment

**School food opportunities.** We adapted a 12-item scale from a previously tested scale used by Kubik and colleagues (7) to serve as the independent variable to determine the frequency of the availability of school food. For each item, 6 response categories represented days per week and ranged from 0 to 5 days. Responses were coded from 0 to 5. Items were summed; a high score indicated more school food opportunities. The Cronbach α was 0.82.

**Key informant interviews.** Trained study staff conducted in-person semistructured interviews with a key staff member from each school (n = 6). In addition to general school-related questions, the interview included questions about the food environment, such as school-wide food practices and policies and school food options for students. Interviews took about 30 minutes to complete.

**Vending machine and school store opportunities.** Trained study staff completed an inventory of all items sold in vending machines and school stores. Information collected on all foods for sale included brand name, package size, number of servings per package, calories per serving, total calories, grams of fat per serving, and total fat. All items were separated into 2 categories, foods and beverages to promote and to limit, under guidelines adapted from criteria published by the Alliance for a Healthier Generation (22) for high schools. Foods having more than 200 calories per package and beverages that were not 100% juice and that contained more than 66 calories per 8-oz serving were considered foods and beverages to limit.

### Statistical analysis

We used descriptive statistics and mixed-model analysis of variance to assess the association between students' dietary behaviors and the scale representing school food opportunities. Continuous dependent variables (student intake of all 3 categories of sugar-sweetened beverages and fruits and vegetables) were positively skewed with Gaussian distributions; therefore, the models were appropriately adjusted with square root transformations, and statistics from these models were used to determine statistical significance. However, mean servings were generated and were reported on the natural scale (untransformed) because they are easier to interpret. The school variable was included in the model as a random effect, accounting for the additional component of variance associated with a cluster sampling design in which observations from students within the same schools may be correlated (23). We used PROC MIXED procedures because the outcome variables were continuous. All the models were adjusted for the potential confounders of sex, age, race/ethnicity, and SES. Age- and sex-adjusted body mass index percentile, calculated from measured height (cm) and weight (kg), was tested as a potential confounder; however, it was not included in the final model because it did not significantly change the associations between the scale and the outcome variables. The level of significance was set at *P* < .05. Analyses were conducted by using SAS statistical software, version 9.1 (SAS Institute, Inc, Cary, North Carolina).

## Results

Among this sample of students, 52% were male, 63% were younger than 18 years (mean, 17.2 y; range, 14.0-19.9 y), 61% were minorities (mean, 62%; range, 31%-96%), and 60% received free/low-cost lunches (mean, 61%; range, 40%-96%). On average, students consumed regular soda more than 10 times a week and visited a fast-food restaurant about 3 times a week (Table 1). In our key informant interview, we found that 4 of the 6 schools did not have wellness advisory councils, and, of the 3 schools that had either vending machines or school stores, none had policies for the food sold in these places (Table 2). More than 60% of the foods and beverages sold in vending machines and school stores were categorized as foods and beverages to limit.

Student reporting of food opportunities during the school day showed that the most frequent food opportunities were getting lunch at a fast-food restaurant (76%), drinking (75%) and eating (70%) in the classroom, and drinking in the school hallways (70%) (Table 3). The results of the multivariate models between each dietary behavior and the food opportunities scale (Table 4) indicate that students who reported using the food opportunities more frequently during the school week had higher consumption of regular soda, sports drinks, other sugar-sweetened beverages, high-fat foods, and fast-food restaurant use. There were no significant associations between the scale and fruit and vegetable consumption.

## Discussion

In our study, alternative high school students reported having multiple food opportunities during the school day, and more frequent food opportunities were associated with higher consumption of sugar-sweetened beverages, high-fat foods, and fast-food restaurant use but were not associated with fruit and vegetable consumption. The results of this study add to an increasing body of research that supports a link between the school food environment and students' dietary practices (4,5,24) and provide support for school policies that limit access to high-calorie, low-nutrition foods (25).

Although all 6 study schools participated in the US Department of Agriculture federally regulated NSLP, half of the schools had an open-campus policy and a third allowed students to leave campus during other periods. Compared with a sample of students predominantly from large suburban traditional high schools, the students in this study more frequently ate lunch at fast-food restaurants (76% vs 18%), purchased lunch at convenience stores (59% vs 8%), and bought food from vending machines (56% vs 43%) at least 1 or 2 times per week (4). Results from national studies have indicated that school food practices limiting access to competitive food sources were associated with up to a 90-calorie reduction in energy intake from sugar-sweetened beverages during the school day among middle or high school students (25). Considering the excess of unhealthful items sold in competitive sources in our schools, limiting the availability of these foods could potentially lower energy intake.

The school food environment is a matter of national concern because of the obesity epidemic among youth (26). As a result, national efforts have focused on creating a more healthful food environment through better school health and nutrition assessments and policies (2,17,24). Furthermore, the Institute of Medicine report of 2007 includes recommendations on the nutrient quality of competitive foods sold at schools (27). Findings from the School Health Policies and Programs Study (SHPPS) 2006 suggest that progress has been made toward a healthier school food environment (24), including higher participation in the NSLP and lower à la carte sales (28). However, the SHPPS focused only on traditional public schools. Results from national studies have shown that Hispanic and African American high school students consume energy-dense foods more frequently than their white counterparts do (25). Considering the high rates of obesity among minority youth and those living below the federal poverty level (26), collecting national data on alternative school health programs and policies, including the food environment, would provide important information to help design health programs to meet the unique needs of this population.

This study had strengths and limitations. The strengths include a diverse sample of adolescents with respect to sex, race/ethnicity, and SES, and the use of measures that have been previously tested in other adolescent populations. This study is the first to examine associations between school food opportunities and dietary behaviors among alternative high school students. Even though the student participation rate was lower than expected, the demographic distribution of our sample closely resembles the demographic characteristics of alternative high school students in our study schools and national studies (29). Limitations also include the cross-sectional nature of the study that considers only the associations between the school food opportunities and student dietary behaviors. Previous research has shown that, compared with 24-hour dietary recalls, the 6-item questionnaire used to measure fruit and vegetable consumption may underestimate the prevalence of fruit and vegetable intake among urban adolescents; however, it performed equally to the Harvard Food Frequency Questionnaire (21). The dietary measures include foods eaten during the entire day; therefore, some eating behaviors may be the result of factors outside of the school environment.

Our findings indicate that the students in our alternative schools have multiple food opportunities during the school day, which is concerning, considering the high enrollment of youth who are at risk for obesity and related health outcomes. To achieve effective outcomes in preventing chronic disease, intervention programs need to target youth who have the highest health-risk behaviors and to tailor these programs to meet their needs. Having data available on alternative high schools and their students will enable researchers to design more effective school health and nutrition interventions. Schools should increase the availability of more healthful foods and beverages, implement closed-campus policies, and improve the quality of school meals to encourage more students to participate in the NSLP. The success of these interventions can be improved by implementing principles from social marketing (30). After having a clear understanding of the target audience, efforts can focus on offering healthful foods that taste good, are priced appropriately, are located conveniently, and are promoted through venues used by these students.

## Acknowledgments

This research was supported by a grant from the National Institutes of Health, National Institute of Diabetes and Digestive and Kidney Diseases, R21DK072948. It was also supported in part by the Adolescent Health Protection Program grant no. T01-DP000112 from the Centers for Disease Control and Prevention. We gratefully thank the school staff and students who participated in the Team COOL pilot study.

## Figures and Tables

**Table 1:** Dietary Behaviors of Alternative High School Students (n = 145), Minneapolis/Saint Paul, Minnesota, 2006

Children's** Dietary Behaviors**	Mean Intake (Range)
Regular soda (times/wk)	10.5 (0-35)
Sports drinks (times/wk)	4.5 (0-35)
Other sugar-sweetened beverages^a^ (times/wk)	7.5 (0-35)
High-fat food intake (times/wk)	26.1 (4.2-64.5)
Fruit and vegetable intake (servings/d)	3.6 (0-24)
Fast-food restaurant use (times/wk)	2.8 (0-8)

a Other sugar-sweetened beverages include Kool-Aid, fruit drinks, lemonade, and energy drinks.

**Table 2:** Food-Related Policies and Practices in 6 Alternative High Schools, Minneapolis/Saint Paul, Minnesota, 2006^a^

Characteristic	Yes, (n)	No, (n)
**School food-related policies**		
Health or wellness advisory council at school	2	4
Policy about the nutrient quality of food sold in vending machines^b^	0	3
Policy about the nutrient quality of food sold in school stores^c^	0	3
**Food-related practices**		
Leave school grounds during lunch	3	3
Leave school grounds during other periods	2	4
Have food and beverages in the classroom	6	0
Have food and beverages in the school hallways	5	1

a Food-related policies and practices were reported by 1 key informant at each school.

b Three schools did not have vending machines.

c Three schools did not have school stores.

**Table 3:** Percentage of Alternative High School Students Reporting Food Opportunities During the School Day, Minneapolis/Saint Paul, Minnesota, 2006

**The School Food Opportunities Scale** a	Days/Week (%)			

Scale Items^b^	0	1-2	3-4	5
During a normal school week, how many days per week do you . . .				
Get lunch at a fast food restaurant	24	44	24	8
Get lunch at a convenience store, gas station, or concession stand	41	38	15	6
Bring lunch from home	84	13	3	0
Get food from a school vending machine or school store	45	31	19	6
Get drinks from a school vending machine or school store	38	29	24	9
Get food or drinks from a vending machine not at school	66	21	10	3
Eat in the hallways at school	45	22	23	10
Eat in the classrooms at school	30	32	23	15
Drink in the hallways at school	30	20	28	22
Drink in the classrooms at school	26	18	34	23
Get food as an incentive or reward from school staff	75	19	4	2
Eat "free food" brought to school by school staff	84	10	2	5

a Cronbach α: 0.82.

b The scale was adapted from a previously tested scale used by Kubik et al (7). It covers any food sources other than the national school breakfast and lunch programs.

**Table 4:** Multivariate Associations Between the Scale Representing Food Opportunities During the School Day and Selected Dietary Behaviors Among Alternative High School Students^a^, Minneapolis/Saint Paul, Minnesota, 2006

Dietary Behaviors	Food Opportunities During the School Day^b^	

	Estimate^b^,^c^ (95% CI)	*P* Value^d^
Regular soda, times/wk (n = 142)	0.24 (0.08-0.39)	<.001
Sports drinks, times/wk (n = 143)	0.23 (0.12-0.34)	<.001
Other sugar-sweetened beverages^e^, times/wk (n = 142)	0.19 (0.06-0.33)	.01
High-fat food intake, times/wk (n = 141)	0.36 (0.18-0.55)	<.001
Fruit and vegetable intake, servings/d (n = 141)	0.05 (−0.011-0.12)	.13
Fast-food restaurant use, times/wk (n = 139)	0.08 (0.06-0.10)	<.001

Abbreviation: CI, confidence interval.

a Sample size varies across models because of missing values.

b A 12-item scale (eg, getting food or drinks from vending machines).

c Each model includes the scale and is adjusted for sex, age, race/ethnicity, and socioeconomic status.

d The square-root transformed outcome variables are used to determine the *P* values.

e Other sugar-sweetened beverages include Kool-Aid, fruit drinks, lemonade, and energy drinks.

## References

[1] Gleason P, Suitor C (2001). Food for thought: children's diets in the 1990s.

[2] Gordon A, Fox MK (2007). US Department of Agriculture, Food and Nutrition Service, Office of Research, Nutrition, and Analysis, School Nutrition Dietary Assessment Study-III summary of findings.

[3] Story M, Neumark-Sztainer D, French S (2002). Individual and environmental influences on adolescent eating behaviors. J Am Diet Assoc.

[4] Neumark-Sztainer D, French SA, Hannan PJ, Story M, Fulkerson JA (2005). School lunch and snacking patterns among high school students: associations with school food environment and policies. Int J Behav Nutr Phys Act.

[5] Kubik MY, Lytle LA, Hannan PJ, Perry CL, Story M (2003). The association of the school food environment with dietary behaviors of young adolescents. Am J Public Health.

[6] French SA, Harnack L, Jeffery RW (2000). Fast food restaurant use among women in the Pound of Prevention study: dietary, behavioral and demographic correlates. Int J Obes Relat Metab Disord.

[7] Kubik MY, Lytle LA, Story M (2005). Schoolwide food practices are associated with body mass index in middle school students. Arch Pediatr Adolesc Med.

[8] Lehr CA, Lange CM Alternative schools and the students they serve: perceptions of state directors of special education. Policy research brief.

[9] Hoffman L Numbers and types of public elementary and secondary schools from the common core of data: school year 2006–07 (NCES 2009-304rev).

[10] Kleiner B, Porch R, Farris E Public alternative schools and programs for students at risk of education failure: 2000-01 (NCES 2002-004).

[11] Grunbaum JA, Lowry R, Kann L (2001). Prevalence of health-related behaviors among alternative high school students as compared with students attending regular high schools. J Adolesc Health.

[12] Kubik MY, Davey C, Fulkerson J, Sirard J, Story M, Arcan C (2009). Alternative high school students: prevalence and correlates of overweight. Am J Health Behav.

[13] Arcan C, Kubik MY, Fulkerson JA, Story M (2009). Sociodemographic differences in selected eating practices among alternative high school students. J Am Diet Assoc.

[14] Sirard JR, Kubik MY, Fulkerson JA, Arcan C (2008). Objectively measured physical activity in urban alternative high school students. Med Sci Sports Exerc.

[15] Kubik MY, Lytle L, Fulkerson JA (2004). Physical activity, dietary practices, and other health behaviors of at-risk youth attending alternative high schools. J Sch Health.

[16] Kubik MY, Lytle L, Fulkerson JA (2005). Fruits, vegetables, and football: findings from focus groups with alternative high school students regarding eating and physical activity. J Adolesc Health.

[17] Healthy schools, local wellness Healthy schools, local wellness policy. US Department of Agriculture, Food and Nutrition Service.

[18] Minnesota Department of Education.

[19] Neumark-Sztainer D, Story M, Hannan PJ, Rex J (2003). New Moves: a school-based obesity prevention program for adolescent girls. Prev Med.

[20] Block G, Gillespie C, Rosenbaum EH, Jenson C (2000). A rapid food screener to assess fat and fruit and vegetable intake. Am J Prev Med.

[21] Field AE, Colditz GA, Fox KM, Byers T, Serdula M, Bosch RJ (1998). Comparison of 4 questionnaires for assessment of fruit and vegetable intake. Am J Public Health.

[22] Alliance for a Healthier Generation.

[23] Murray DM (1998). Design and analysis of group-randomized trials.

[24] O'Toole TP, Anderson S, Miller C, Guthrie J (2007). Nutrition services and foods and beverages available at school: results from the School Health Policies and Programs Study 2006. J Sch Health.

[25] Briefel RR, Crepinsek MK, Cabili C, Wilson A, Gleason PM (2009). School food environments and practices affect dietary behaviors of US public school children. J Am Diet Assoc.

[26] Ogden CL, Carroll MD, Flegal KM (2008). High body mass index for age among US children and adolescents, 2003-2006. JAMA.

[27] (2007). Institute of Medicine. Nutrition standards for foods in schools: leading the way toward healthier youth. Report brief.

[28] Wojcicki JM, Heyman MB (2006). Healthier choices and increased participation in a middle school lunch program: effects of nutrition policy changes in San Francisco. Am J Public Health.

[29] Grunbaum JA, Kann L, Kinchen SA, Ross JG, Gowda VR, Collins JL (1999). Youth Risk Behavior Surveillance — National Alternative High School Youth Risk Behavior Survey, United States, 1998. MMWR CDC Surveill Summ.

[30] Grier S, Bryant CA (2005). Social marketing in public health. Annu Rev Public Health.

